# APX005M, a CD40 agonist antibody with unique epitope specificity and Fc receptor binding profile for optimal therapeutic application

**DOI:** 10.1007/s00262-020-02814-2

**Published:** 2021-01-03

**Authors:** Erin L. Filbert, Pia K. Björck, Minu K. Srivastava, Frances R. Bahjat, Xiaodong Yang

**Affiliations:** Apexigen, Inc, 75 Shoreway Road, Suite C, San Carlos, CA 94070 USA

**Keywords:** CD40, Fc receptors, Immunotherapy, Antigen-presenting cell, Agonist, Immune checkpoint activator

## Abstract

Targeting CD40 with agonist antibodies is a promising approach to cancer immunotherapy. CD40 acts as a master regulator of immunity by mobilizing multiple arms of the immune system to initiate highly effective CD8 + T-cell-mediated responses against foreign pathogens and tumors. The clinical development of CD40 agonist antibodies requires careful optimization of the antibody to maximize therapeutic efficacy while minimizing adverse effects. Both epitope specificity and isotype are critical for CD40 agonist antibody mechanism of action and potency. We developed a novel antibody, APX005M, which binds with high affinity to the CD40 ligand-binding site on CD40 and is optimized for selective interaction with Fcγ receptors to enhance agonistic potency while limiting less desirable Fc-effector functions like antibody-dependent cellular cytotoxicity of CD40-expressing immune cells. APX005M is a highly potent inducer of innate and adaptive immune effector responses and represents a promising CD40 agonist antibody for induction of an effective anti-tumor immune response with a favorable safety profile.

**Supplementary Information:**

The online version contains supplementary material available at 10.1007/s00262-020-02814-2.

## Introduction

Targeting of immune checkpoint inhibitor (ICI) proteins including cytotoxic T-lymphocyte-associated protein 4 (CTLA-4) and program cell death 1 (PD-1) or its ligand, PD-L1, is able to partially reverse cancer immunosuppression of tumor-specific cytotoxic effector cells by unleashing them to kill tumor cells. However, durable responses to treatment are achieved only in a minority of cases. Most patients never respond to treatment or have an initial response followed by tumor progression which highlights the need for new immunotherapies such as immune agonists.

CD40 remains an attractive target for cancer immunotherapy, representing a key component of both the innate and adaptive immune response. CD40 is a member of the TNF receptor superfamily expressed on a variety of immune cells, including B cells, dendritic cells, macrophages and monocytes [1]. CD40 agonist antibodies can potently activate anti-tumor immunity via multiple mechanisms. First, CD40-mediated activation of antigen presenting cells (APCs) is a prerequisite for T-cell activation and thus can promote the generation of tumor-specific cytotoxic T cells even in the absence of CD4 T-cell help [2–4]. Second, CD40 agonists can directly activate macrophages to infiltrate and deplete tumor stroma, converting ‘cold’ tumors into ‘hot’ tumors [5]. Finally, CD40-activated APCs produce IL-12 and IL-15, cytokines that are essential for NK cell-mediated killing of tumor cells [6].

Preclinical mouse models have provided significant rationale for the clinical development of CD40 agonist antibodies as single agent therapies or in combination with standard of care in a variety of tumor types [7–9]. In a mouse model of bladder cancer, CD40 agonist antibodies show single-agent activity when administered either systemically or locally [10]. CD40 agonist antibodies induce tumor regression in mouse models of pancreatic cancer as a single-agent [5] and when combined with ICI [11, 12]. Numerous vaccine strategies for cancer therapy are currently being evaluated [13], and the addition of CD40 agonist antibodies enhances the activity of tumor vaccines in nearly every case studied [14–16] providing strong rationale for combination.

For almost a decade, CD40 agonist antibodies have been investigated clinically in a range of tumor types with compelling initial results [7, 17] [18] [19]. Clinical efficacy in melanoma was demonstrated in front line therapy after only a single injection of the CD40 agonist CP-870,893 [20, 21]. In a phase I study combining CD40 agonist and CTLA-4 blockade in patients with metastatic melanoma, long-term protection was observed with 9 of 24 patients with survival currently beyond 3 years (18.2% PR plus 9.1% CR = ORR is 27.3%; mean OS 23.6 months) [21]. While encouraging data have been observed, on-target immune-related adverse events (IRAEs) including cytokine release syndrome and liver enzyme elevation have historically hampered the enthusiasm for further clinical development. Preclinical studies have shed light on strategies that could fine-tune CD40 agonist antibodies to improve safety profiles and enhance efficacy.

Epitope specificity and isotype of CD40 agonist antibodies contribute to their function and clinical activity [14, 22–24]. CD40 agonist antibodies are able to induce CD40 signaling to various degrees whether or not they block natural ligand binding. Studies using anti-mouse CD40 antibodies have shown that stronger agonist activity correlated with blockade of CD40 ligand (CD40L) binding, likely because these antibodies better recapitulate the natural ligand’s ability to activate APCs [25]. Moreover, the anti-tumor efficacy of CD40 agonists in mouse models was greatly improved by engineering Fc variants with enhanced binding to FcγRIIb, including the S267E variant that confers 30-fold increased binding affinity to FcγRIIb [14, 26, 27]. Enhanced binding to FcγRIIb may contribute to enhanced efficacy by mediating crosslinking of the antibody and clustering of CD40 receptors in a way that mimics the natural CD40L interaction and by inhibiting ADCC activity on CD40-expressing APCs due to the lack of binding to FcγRIIIa. Importantly, CD40 agonists that depend on FcγRIIb crosslinking should have a more favorable safety profile because FcγRIIb-mediated crosslinking is most prevalent in tumor tissues and tumor-draining lymph nodes but limited in the circulation which could lead to less systemic APC activation and cytokine release.

We generated a panel of high affinity CD40 antibodies using the APXiMAB™ platform. APX005M was selected based on its potent agonist activity and binding to the CD40L binding site. APX005M was further engineered to include the S267E mutation which significantly enhanced its agonistic activity and minimized ADCC effector function. We show here that APX005M’s unique structural attributes give it desirable biological and pharmacological activities which are differentiated from other CD40 agonists in the clinic. Thus, APX005M represents a promising immune checkpoint activator for cancer immunotherapy. Multiple clinical trials are being conducted with APX005M alone and in combination with other therapies across several tumor types [28, 29].

## Materials and methods

### CD40 antibodies

APX005M is a humanized IgG1κ antibody generated by Apexigen using its APXiMAB™ technology consisting of a rabbit hybridoma platform and mutational lineage-guided (MLG) humanization method [30–32]. A point mutation was introduced in the Fc-domain at position 267 from serine to aspartic acid (S267E mutation). A version of APX005M lacking S267E mutation (APX005), as well as recombinant analogues of anti-CD40 agonistic antibodies CP-870,893, SGN-40 and ADC-1013 were synthesized based on published data [33–35]. F(ab’)2 fragments of APX005M were generated by pepsin digest (Rockland Immunochemicals).

### Reagents

Human cytokines were purchased from Peprotech (Rocky Hill, NJ). CD40-his and CD40L were purchased from Cedarlane (Burlington, NC). Isotype and flow cytometry antibodies (CD4-PE, CD8-PE, CD40-APC, CD14-FITC, CD14-PECy7, HLA-DR APC-Cy7 or FITC, CD11c-Alexa Fluor 488, CD86-PE) were obtained from Biolegend or Becton Dickinson (San Jose, CA; CD80-APC, CD83-PE). Rituximab was purchased from InvivoGen (San Diego, CA).

### CD40 binding assay

For B cell binding, 1 × 10^6^ human peripheral blood mononuclear cells (PBMC) per 96-well were incubated with increasing concentrations of CD40-targeted antibodies at 4 °C for 1 h, then washed and stained at 4 °C for 30 min with anti-human CD20-FITC to gate the human B-cell population and with goat F(ab′)_2_ anti-human Fcγ PE to detect anti-CD40 antibodies. For DC binding, monocyte-derived DC (moDC) were generated as follows: human PBMC were isolated from buffy coats (Stanford Blood Center, Palo Alto, CA) using Ficoll® Hypaque (GE Healthcare, Pittsburgh, PA) density gradient centrifugation. After washing, CD14 + monocytes were isolated using magnetic beads (Miltenyi Biotech, Auburn, CA). Cells were cultured for 7 days in RPMI-1640 with 10% fetal bovine serum (Hyclone, GE Healthcare), penicillin–streptomycin, and 2 mM L-glutamine (Life Technologies, Grand Island, NY) with 100 ng/mL GM-CSF and 50 ng/mL IL-4. DCs were stained as described above. All samples were analyzed on a MACSQuant® flow cytometer. For each antibody titration, results are expressed as mean fluorescence intensity (MFI) for CD40 on CD40^+^ cells.

### APC activation

Freshly isolated CD19^+^ human B cells were incubated at 37 °C in 96-well round-bottom plates (1 × 10^5^ cells/100 µL) in the presence of CD40 agonist antibodies. After 48 h, cells were washed and stained with mouse anti-human CD86 PE. For HLA-DR expression on B cells, 200 µL of human whole blood was stimulated with APX005M in 96-well round bottom plates for 24 h then stained with anti-human HLA-DR FITC antibody. RBCs were then lysed with ACK lysis buffer and re-suspended in PBS + 0.5% BSA + 2 mM EDTA. All samples were analyzed on the MACSQuant® flow cytometer. For DC activation, moDC were generated as described above and 5 × 10^5^ cells per well were cultured in 96-well plates in the presence of CD40 agonist or control antibodies for 48 h. Supernatants were harvested and assayed for IL-12p70 by ELISA (R&D Systems) and upregulation of CD86 was determined as described above.

### Epitope mapping of APX005M

Chemically linked peptides on scaffolds (CLIPS) technology and peptide arrays were used for epitope mapping (Pepscan Presto, Lelystad, The Netherlands) [36]. In brief, peptide cysteine residues were coupled to CLIPS templates on the peptide array. After incubation for 30–60 min, the peptide array was washed with H_2_O and sonicated in the presence of 1% sodium-dodecyl sulphate-1% 2-mercaptoethanol in PBS at 70 °C for 30 min followed by sonication in H_2_O for 45 min. Antibody binding to the peptides was examined by ELISA. Peptide arrays were incubated with primary antibody overnight at 4 °C. After washing, peroxidase-conjugated secondary antibody was added and incubated for 30 min and, after additional washing, ABTS peroxidase substrate and 3% H_2_O_2_ was added. Color change was quantified after 1 h using a CCD camera and an image processing system.

### CD40L blocking assay

ELISA plates were coated with 0.5 µg/ml CD40-his overnight. After blocking with 1% BSA in PBS, serial dilutions of APX005M or isotype control were added and plates were incubated for 1 h at room temperature. 3 µg/ml CD40L was added and the plate was incubated for 1 h. After washing, mouse anti-human CD40L (R&D Systems) was added and incubated for 1 h. Donkey anti-mouse IgG (Jackson ImmunoResearch) was added for one hour and detected using TMB substrate (Kirkegaard & Perry, Gaithersburg, MD) read at OD 450 nm.

### Fc receptor binding assay

CHO cells were transfected with cDNAs encoding human Fc receptors. Expression was confirmed by flow cytometry (Eureka Therapeutics, Emeryville, CA). Cells were detached using 2.5 mM EDTA in PBS, washed and added to 96-well plates. Serial dilutions of antibodies were added and samples incubated on ice for 1 h. After washing, PE-conjugated F(ab)’2 fragment of goat anti-human IgG (Jackson ImmunoResearch) was added and cells were incubated for 30 min on ice and analyzed by FACS. Saturation binding curves were generated using nonlinear regression and one-site binding model provided in GraphPad Prism software (GraphPad Software, San Diego, CA) to derive KD values.

### Antibody-dependent cell cytotoxicity assay (ADCC)

Daudi (ATCC® CCL-213™) B cells (40,000 per well) in RPMI-1640 with 10% ultra-low IgG were added to 96-well plates. Serial dilutions of indicated antibodies were added. CD56 + CD16 + NK cells were obtained from PBMC using magnetic bead separation (Miltenyi Biotech) and were added to plates (200,000 per well) resulting in an effector to target (E:T) ratio of 5:1. Plates were incubated for 4 h at 37 °C. After incubation, supernatant was transferred to 96-well plates and CytoTox® substrate mix added to each well (Promega; Madison, WI). Plates were incubated in the dark for 30 min then read at OD = 492 nm using SpectraMax® M3 spectrophotometer. Percent cytotoxicity of target cells was reported from triplicate measures and at least three donors as % cytotoxicity = (effector (spontaneous)—target (spontaneous))/(target (maximal)—target (spontaneous))*100, where medium background was subtracted from all values.

### Mixed lymphocyte reaction (MLR)

Human T cells were labeled with eFluor 670 (eBioscience) and cultured (2 × 10^5^ cells per well) with allogeneic moDC (5 × 10^4^ cells per well) in flat-bottomed 96-well plates. moDCs were pre-activated with CD40 agonistic or control antibodies for 24 h prior to the addition of T cells. After 6 days of co-culture, supernatants were harvested and assessed for IFN-γ by ELISA (R&D Systems). To measure T-cell proliferation, cells were stained with anti-CD8 PE antibodies and T-cell divisions were evaluated using a MACS Quant® analyzer.

### Viral antigen recall assay

PBMC were suspended in culture media and plated in 96-well plates at 10^6^ per well. EBV peptides (Miltenyi Biotech; 130–099-764) were added at a final concentration of 1μg/mL. CD40 agonist antibodies were added and plates were incubated at 37˚C. After 5 days of culture, supernatants were removed and assayed for IFN-γ by ELISA.

### Ex vivo tumor experiments

Tumor biopsies from consented patients undergoing surgery for human non-small cell lung cancer (NSCLC) were harvested and dispersed into single cell suspensions (Nilogen Oncosystems, Tampa, FL). APX005M (67 nM) or vehicle was added and cultures were incubated for 5 days. T cells were stained with antibodies to CD3 and Ki67 along with viability dye, and analyzed by flow cytometry. Gates were set to exclude dead cells and debris, and the number of CD3 + Ki67 + T cells was determined. Data are representative of three donors.

### APX005M washout assay

Human B cells were incubated with APX005M or control antibody for 7 days. In indicated samples, antibodies were washed out after 24 h. Following 7 day incubation, B cells were harvested and expression of CD86 and HLA-DR was assessed by flow cytometry as described above.

### CD40 receptor occupancy and maximal B-cell activation

A weighed least-square procedure in Phoenix WinNonlin 6.4 (Pharsight, Inc. Mountain View, CA) was used to establish a correlation between CD40 receptor occupancy on B cells and cellular activation according to the equation: *E* = *E*_0_ + *E*_max_*C*^γ^/EC^γ^_50_ + *C*^γ^ where E is the effect, E_0_ is the effect at baseline, E_max_ is the maximum effect and EC_50_ is the concentration at half maximum effect. The predicted CD40 receptor occupancy at maximum B-cell activation (CD86 expression) was derived from this fit.

### Preclinical safety assessment of APX005M

GLP tissue cross-reactivity study on a full panel of normal human and cynomolgus monkey tissues was conducted at Charles River Laboratories, Frederick, MD. APX005M or isotype control was applied at 10 and 50 µg/mL.

Non-human primate (NHP) studies were carried out in cynomolgus monkeys by Charles River Laboratories, Reno, NV. Ten monkeys in each group (2.5–4 kg, five males and five females per group) received a once-weekly intravenous bolus injection of 0.3, 3 or 30 mg/kg body weight of APX005M for 4 weeks. Standard in-life and terminal analyses were conducted, including peripheral lymphocyte analysis by flow cytometry and serum analysis for cytokines (Luminex) and APX005M (antigen capture ELISA). PK analysis was performed by PharmaPolaris International (North Potma, MD).

### Statistical analyses

Statistical analyses were performed using Graph Pad Prism® (Graph Pad Software, San Diego, CA) as indicated.

## Results

### Characterization of APX005M binding specificity and activity

**Fig. 1:**
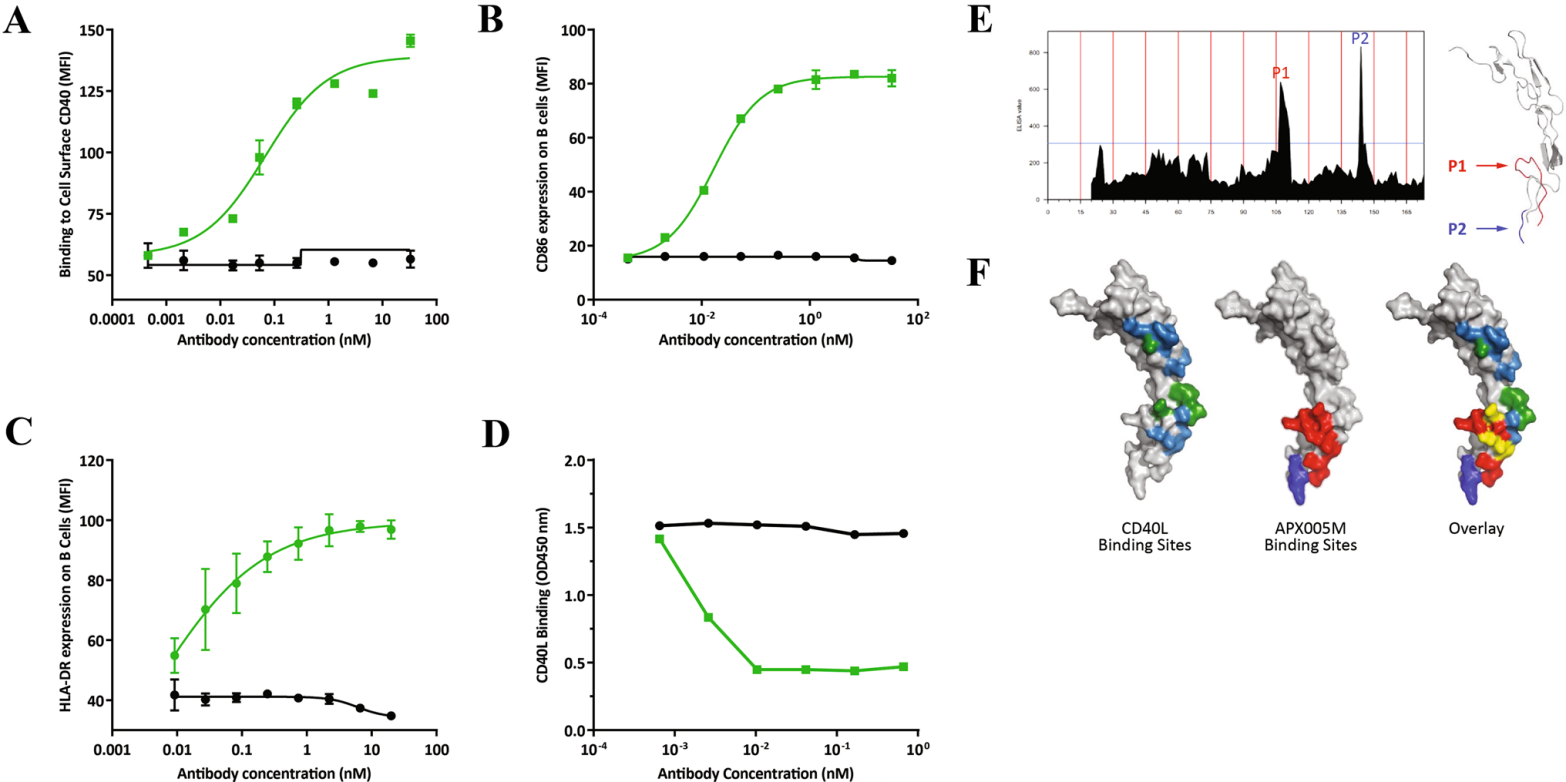
APX005M binds to CD40 with high affinity and potently activates B cells. **a** Binding of APX005M on human B cells was determined by flow cytometry and expressed as mean fluorescent intensity (MFI). B-cell activation was measured by MFI of CD86 expression after 48-h incubation (**b**) or HLA-DR expression after 24 h (**c**). APX005M is labeled with green squares and isotype control by black circles. Experiments were repeated with at least two donors. **d** Competition ELISA showing that APX005M (green squares) but not isotype control (black circles) can compete with CD40L for binding to CD40. **e** ELISA results for 20‐mer overlapping peptides. X‐axis indicates the residue number of the C‐terminal position (i.e._1_EPPTACREKQYLINSQCCSL_20_ is positioned on X‐value 20). Residues _92_TSEACESCVLHRSCSP_107_ (P1) and _122_ICEP_125_ (P2) are visualized on the cartoon rendering of structure 3QD6. (F) Surface rendering of 3QD6 indicating CD40L binding sites (green and light blue), APX005M binding sites (red and purple) and residues identified as overlapping (yellow)

APX005M was selected from a panel of rabbit monoclonal antibodies based on binding specificity for human CD40 and potent bioactivity. APX005M was humanized as previously described [32]. APX005M binds with high affinity (KD = 1.2 × 10^−10^ M) to B-cell-expressed human CD40 (Fig. 1a) and cynomolgus CD40 (KD = 3.7 × 10^−10^ M), but does not recognize rodent CD40 (data not shown). APX005M does not bind closely related TNFR family members, including OX-40, RANK, DR5, TWEAK-R and 4-1BBL (data not shown). The primary mode of action of CD40 agonist antibodies is the activation of APCs. In vitro APX005M stimulation of human B cells up-regulated immune co-stimulatory molecules CD86/B7.2 (Fig. 1b) and MHC II (Fig. 1c).

Previous studies using anti-mouse CD40 antibodies have shown agonist activity positively correlates with their ability to inhibit CD40L binding [25]. Using a competitive ELISA, we show that APX005M blocks binding of CD40 to CD40L (Fig. 1d) via a unique epitope identified using a comprehensive peptide array consisting of 20-mer overlapping peptides derived from CD40. Based on the published structure of CD40 [37], 12 potential binding-hotspots were identified for single-position mutational analysis and were included in our peptide array. Evaluation of these peptides revealed that APX005M binds two distinct regions: _92_TSEACESCVLHRSCSP_107_ (P1) and _125_PCPVGFFSNVSSAFEKCHPW_144_ (P2), with the residues _92_TSEACESCVLH_102_ anticipated to be the most relevant for binding (Fig. 1e). Visualization of these areas on the crystal structure of CD40 shows that P1 forms a looped structure facing the CD40L trimer (Fig. 1f). Of the identified binding residues, four (T_92_, E_97_ and _100_VL_102_) are known to be contact residues between CD40 and CD40L [37]. These data indicate that APX005M binds to a unique epitope on CD40 that overlaps with the ligand binding site and effectively competes for binding with CD40L.

### APX005M engages FcγRIIb and requires crosslinking for optimal activity

**Fig. 2:**
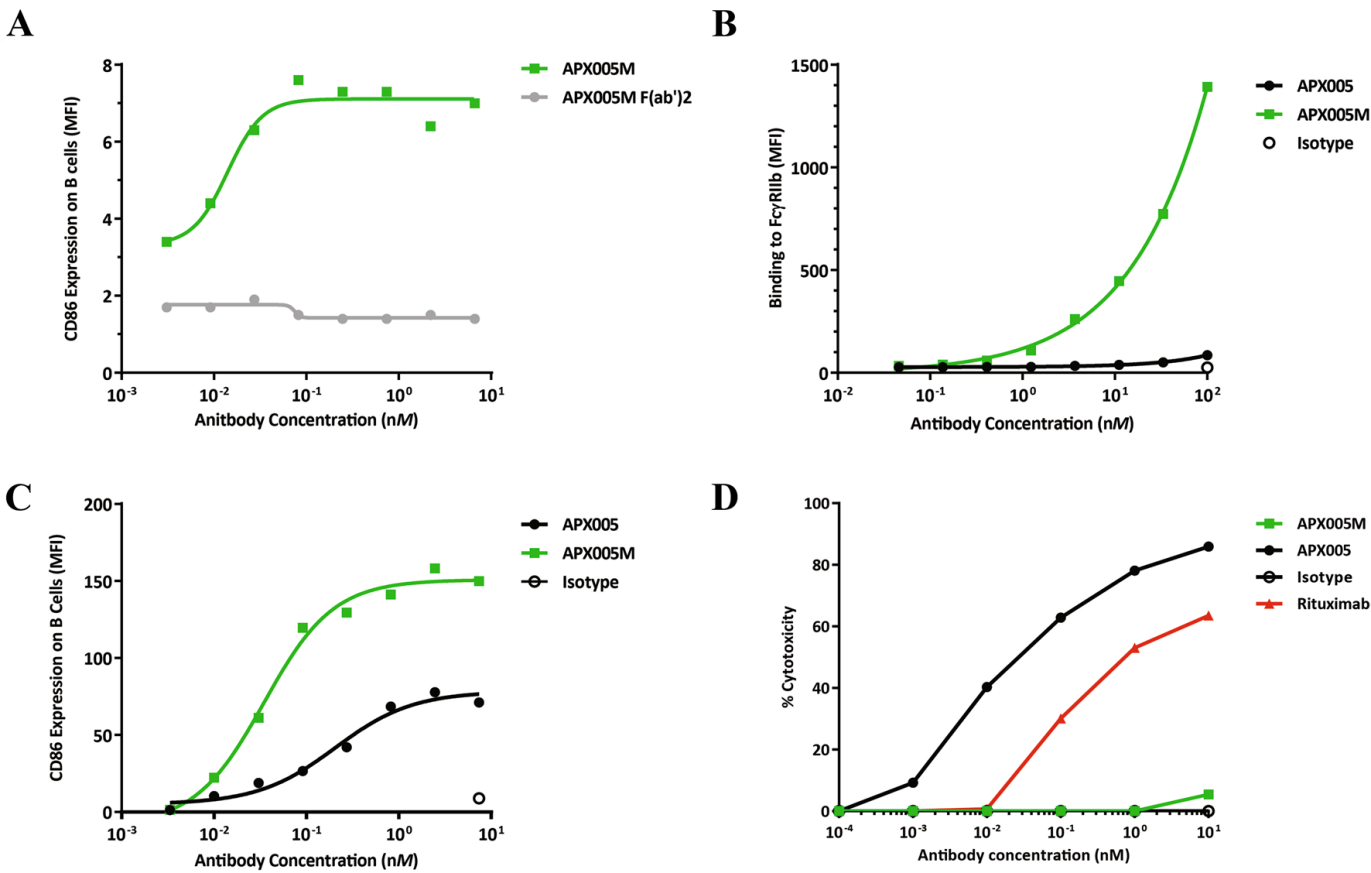
APX005M agonist activity requires FcR-mediated crosslinking. **a** Activation of B cells after 48 h by APX005M intact (green squares) or APX005M F(ab’)2 fragments (gray circles) as measured by MFI of CD86 expression. Data are representative of two donors. **b** Binding of APX005M (green squares), APX005 (black circles) and isotype control (open circle) to human FcγRIIb—expressing CHO cells. **c** Activation of B cells by APX005M (green squares), APX005 (black circles) and isotype control (open circle) for 48 h as measured by MFI of CD86 expression. Data are representative of three donors. **d** NK cell-mediated ADCC activity of APX005M (green squares) and APX005 (black circles) on human Daudi B cells. Rituximab (red triangles) and human IgG1 isotype control (open circles) were used as controls. All conditions were performed in triplicate. Graph is representative of three donors

While APX005M-F(ab’)2 fragments bind CD40 with the same affinity as intact APX005M (data not shown), they fail to stimulate B cells (Fig. 2a) indicating the agonist activity of APX005M is critically dependent upon FcR binding. To increase Fc receptor-mediated crosslinking via FcγRIIb, we engineered the S267E mutation [27] into APX005M with the intention of enhancing agonist activity and improving its safety profile. To determine the effect of the S267E mutation on FcγR binding of APX005M, CHO cell lines expressing individual human Fc receptors were incubated with serial dilutions of APX005M or a version of APX005M with the wildtype IgG1 Fc lacking the S267E mutation (APX005). As shown in Fig. 2b, APX005M strongly bound FcγRIIb (KD = 5.8 × 10^–8^ M), whereas APX005 exhibited negligible binding. APX005M also lost binding to FcγRIIIa but retained binding to other FcγRs in comparison to APX005 (Supplemental Fig. 1). The increase of binding to FcγRIIb resulted in enhanced B cell activation as determined by CD86 upregulation (Fig. 2c).

### APX005M does not mediate antibody-dependent cell cytotoxicity

We assessed the ADCC activity of APX005M alongside APX005 to confirm lack of effector function due to loss of binding to FcγRIIIa. Dose-dependent killing of CD40 + Daudi B-cell targets by CD56 + CD16 + NK cells was evaluated after APX005M or APX005 treatment by monitoring release of lactate dehydrogenase into the culture media. As expected, APX005M was unable to mediate ADCC of CD40-expressing cells (Fig. 2d) as compared to APX005.

### APX005M enhances T-cell responses in vitro

**Fig. 3:**
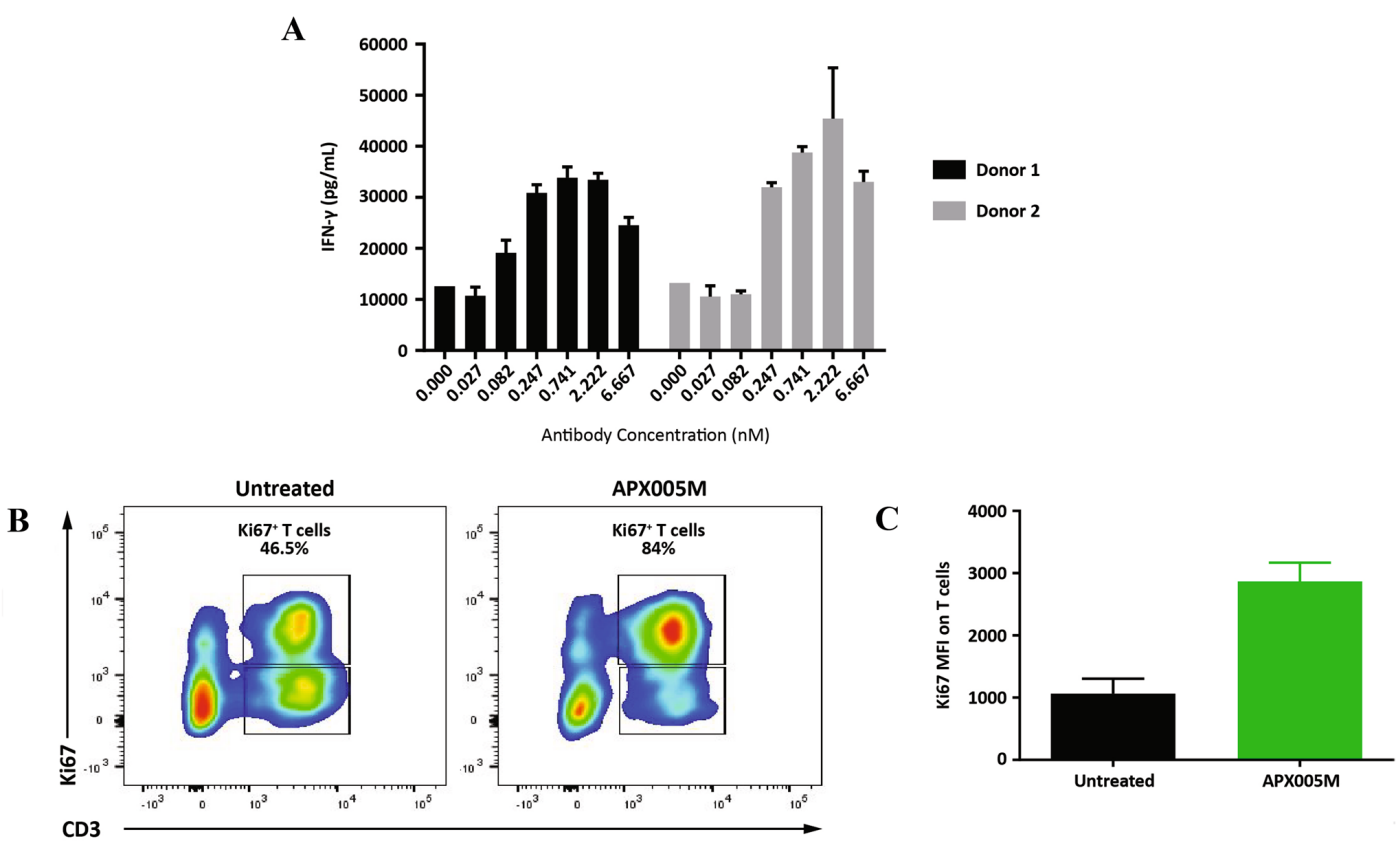
APX005M enhances T-cell responses in vitro. **a** Enhancement of antigen-specific T-cell response measured by IFN-γ production by human PBMCs stimulated with EBV peptides in the presence of indicated concentrations of APX005M for 5 days. Culture supernatants were assayed for IFN-γ in triplicate and data are shown as mean ± SEM. **b** Ex vivo cultures of NSCLC biopsies were treated with APX005M and T-cell proliferation was assessed by Ki67 expression on CD3 + T cells. Representative FACS data are shown in (**c**). Graph depicts MFI of Ki67 on CD3 + cells and represents data from three different NSCLC patients. *p value < 0.01

Since the primary mode of action (MOA) of APX005M is to activate APCs and prime tumor-specific T-cell responses, we performed a viral antigen recall assay using primary human PBMC from donors previously exposed to EBV challenged with EBV antigens. APX005M potently enhanced IFN-γ secretion in a dose-dependent manner in cultures stimulated with EBV antigens (Fig. 3a) supporting this MOA.

To assess the therapeutic potential of APX005M to generate anti-tumor T-cell responses, ex vivo assays from tumor biopsies were performed. Single-cell suspensions containing both tumor cells and tumor-infiltrating immune cells derived from freshly isolated human non-small cell lung cancer (NSCLC) biopsies from consented patients were stimulated with APX005M (67 nM) for 5 days and then T-cell proliferation was determined. As shown in Fig. 3b, c, APX005M significantly enhanced tumor-infiltrating T-cell proliferation. These data suggest APX005M may promote antigen presentation by APCs, leading to the activation of tumor-infiltrating T-cell responses.

### APX005M is functionally distinct from other CD40 agonist antibodies

**Fig. 4:**
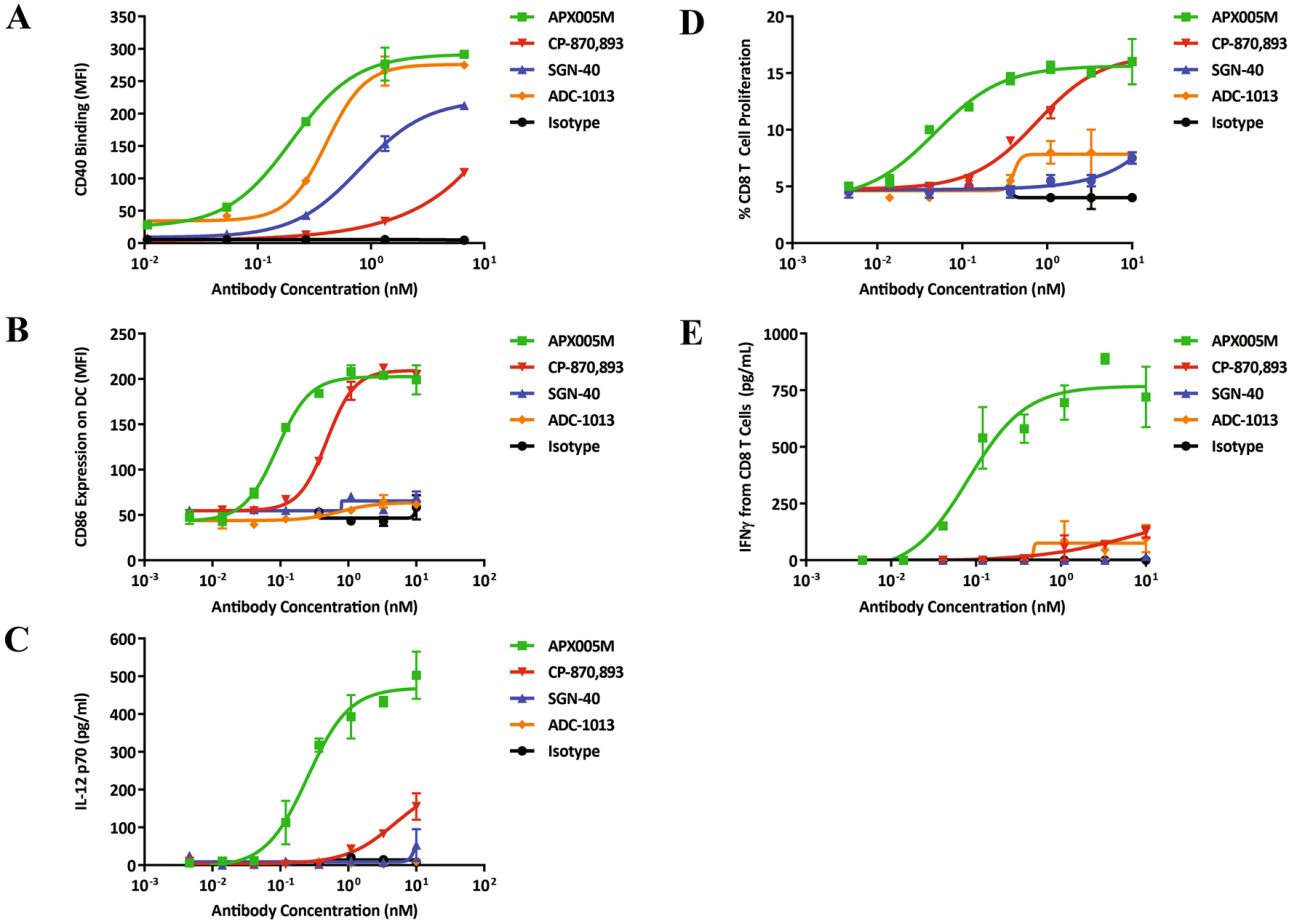
APX005M induces potent activation of dendritic cells. **a** Binding of CD40 agonist antibodies to monocyte-derived DC (moDCs). Activation of moDCs by CD40 agonist antibodies for 48 h was measured by **b** MFI of CD86 expression and **c** IL-12p70 production in culture supernatants. All data are represented as mean ± SEM of triplicate wells and assays were performed in at least two donors. **d** The effect of CD40 agonist antibodies on T cells was assessed in MLR. moDCs were stimulated with serial dilutions of CD40 agonist antibodies for 24 h, and allogeneic eFluor 670-labeled CD8 T cells were added. Cell proliferation indicated by eFluor 670 dye dilution was assessed on day 6 using a MACSQuant Analyzer. IFN-γ secretion was measured from cell supernatants of MLR cultures (**e**). All data are represented as mean ± SEM of triplicate wells and assays were performed in at least two donors

APX005M has unique structural characteristics compared to other reported CD40 agonist antibodies in clinical development. To determine whether these differences impart distinct functionality of APX005M, we compared the biological activity of analogs of CP-870,893, SGN-40 and ADC-1013. Though all antibodies tested showed binding to CD40 on DC (albeit with different affinities) (Fig. 4a), only CP-870,893 analog and APX005M were able to stimulate APCs (Fig. 4b). Interestingly, only APX005M was able to induce substantial secretion of IL-12p70 from human DCs, which is a key functional difference as IL-12 is critical for the activation of naïve T cells (Fig. 4c).

To evaluate whether the observed differences in DC activation for these agonists translate to similar differences in downstream T-cell activation, we performed mixed lymphocyte reactions (MLR) using DC pre-activated with varying concentrations of antibody then co-cultured with allogeneic human CD8 T cells to examine degree of proliferation and IFN-γ induction. APX005M-activated APCs induced proliferation of CD8 T cells (Fig. 4d). CP-870,893 also induced proliferation of T cells, though to a lesser extent than APX005M. In contrast, SGN-40 and ADC-1013 were weak inducers of T-cell proliferation. Interestingly, only APX005M was able to induce significant secretion of IFN-γ (Fig. 4e), correlating with IL-12 secretion from DC (Fig. 4c). Similar results were obtained with CD4 T cells (data not shown). These data suggest that the epitope and Fc mutation of APX005M drive more potent activation of both the innate and adaptive immune responses as compared the other CD40 agonists tested.

### APX005M induces long-lasting APC activation and achieves maximum potency at 10% receptor occupancy

**Fig. 5:**
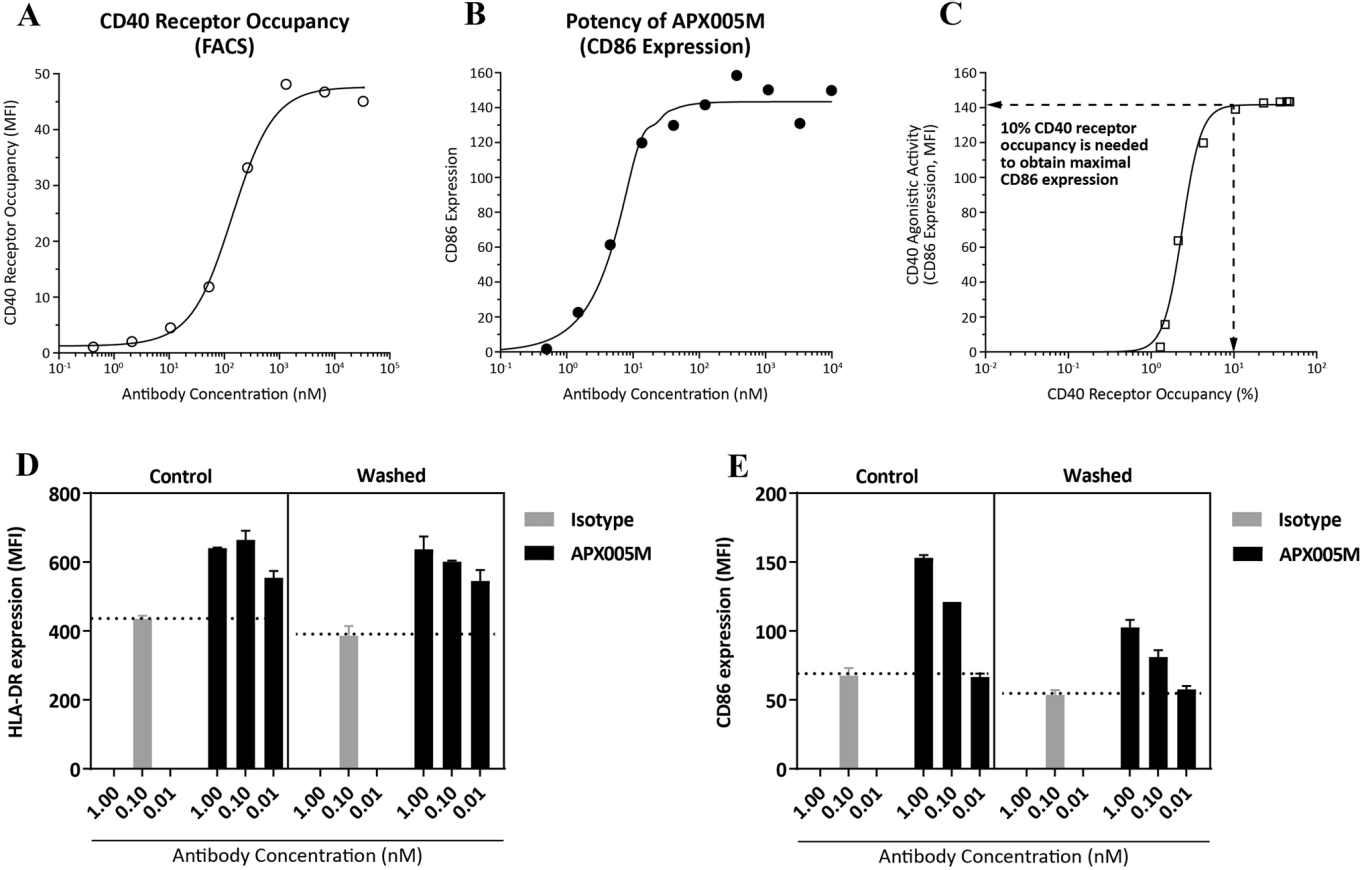
APX005M induces long-lasting APC activation and achieves maximum potency at 10% receptor occupancy. A weighed least-square procedure in Phoenix WinNonlin 6.4 was used to establish a correlation between CD40 receptor occupancy on B cells (**a**) and B-cell activation (**b**). The predicted CD40 receptor occupancy at the predicted maximum B-cell activation as expressed of CD86 expression was derived from this fit (**c**). CD19 + B cells were cultured with indicated concentrations of APX005M or isotype control antibody for 1 week. In washed conditions, antibodies were washed out of the cultures after 24-h incubation. In control conditions, antibodies were maintained throughout culture. At the end of the week, B cells were harvested and expression of HLA-DR (**d**) and CD86 (**e**) was assessed by flow cytometry. Data are presented as mean + SEM and represents at least three different experiments

As shown above, APX005M possesses unique attributes that may translate into better safety and efficacy in patients. To support clinical development for first-in-human studies, we explored the pharmacodynamics of APX005M. We first examined the relationship between receptor occupancy and bioactivity. The dose–response relationship between CD40 receptor occupancy (Fig. 5a) and B-cell activation (Fig. 5b) revealed that only 10% receptor occupancy (1 nM) is sufficient to produce maximum activity (CD86 expression on B cells) (Fig. 5c), suggesting that saturation of CD40 receptors is not required for APX005M to achieve optimal agonistic activity and a low clinical dose may be sufficient to achieve maximal pharmacological effect.

Next, we performed assays to determine the duration of B-cell activation following short-term exposure to APX005M. B cells were stimulated with APX005M or isotype control antibody for 24 h and then antibodies were washed out. After washout, B cells were continuously cultured for 1, 2 or 3 weeks and HLA-DR (Fig. 5d) and CD86 (Fig. 5e) were measured. As a control, B cells were cultured with APX005M or isotype control antibody without washout for 1 week. B cell activation was similar in B cell cultures that had been exposed to APX005M throughout the culture for 1 week to those that had been exposed to APX005M for only 24 h (Fig. 5d, e). B-cell activation was detected up to 3 weeks following 24-h exposure, though not to the same levels as B cells exposed to APX005M throughout the culture (data not shown). These results indicate that short-term exposure to APX005M can induce long-lasting activation of APC which should allow for reduced dosing frequency.

### APX005M pharmacology in non-human primates

A tissue-cross-reactivity study of APX005M was performed on human and cynomolgus macaque tissue panels. Positive staining by APX005M was considered to be generally consistent with known sites of CD40 antigen expression and positive specific staining was seen in multiple cell types including follicular lymphocytes, bone marrow cells, macrophages, endothelial cells and reticular cells in the thymic medulla. Staining was similar between human and cynomolgus tissues which supported use of the cynomolgus monkey as an appropriate species for toxicologic evaluation.

The safety profile of APX005M was thus assessed in a repeat-dose study in cynomolgus monkeys. APX005M was administered IV at 0.3, 3 and 30 mg/kg weekly for 4 weeks (a total of 5 doses). The observed PK properties of APX005M (Table 1) appeared typical of other humanized monoclonal antibodies in monkeys. Increases in the dose of APX005M (0.3 mg/kg to 30 mg/kg) led to dose-proportional increases in* C*_max_ and more than dose-proportional increases in AUC_(0–168)._ The no observable adverse effect level (NOAEL) was determined to be 30 mg/kg.

**Table 1:** Pharmacokinetic parameters of APX005M in Cynomologus Monkeys

Dose (mg/kg)	Gender	Day	*t*_1/2_ (h)	*C*_max_ (µg/mL)	AUC_(0–168)_ (h*mg/mL)	AUC_0-∞_ (h*mg/mL)	*C*_max_/D	AUC_(0–168)_/D
0.3	Female	1	9.89 ± 5.96	5.25 ± 0.424	0.0587 ± 0.0121	0.0587 ± 0.0122	17.5 ± 1.41	0.196 ± 0.0404
		22	19.7 ± 14.4	3.77 ± 2.27	0.0502 ± 0.0207	NA	12.6 ± 7.56	0.167 ± 0.0691
	Male	1	6.79 ± 0.534	4.34 ± 0.762	0.0427 ± 0.00867	0.0427 ± 0.00867	14.5 ± 2.54	0.142 ± 0.0289
		22	21.2 ± 19.3	1.77 ± 1.82	0.0284 ± 0.0263	NA	5.91 ± 6.07	0.0947 ± 0.0876
3.0	Female	1	70.2 ± 23.5	65.7 ± 7.83	3.11 ± 0.769	3.81 ± 1.33	21.9 ± 2.61	1.04 ± 0.256
		22	102 ± 34.7	80.6 ± 11.4	5.53 ± 1.39	NA	26.9 ± 3.80	1.84 ± 0.464
	Male	1	66.2 ± 10.8	81.3 ± 23.2	4.15 ± 0.601	4.95 ± 0.838	27.1 ± 7.74	1.38 ± 0.200
		22	116 ± 23.0	95.4 ± 8.37	6.93 ± 0.747	NA	31.8 ± 2.79	2.31 ± 0.249
30.0	Female	1	106 ± 15.2	812 ± 49.0	49.8 ± 4.61	72.9 ± 9.20	27.1 ± 1.63	1.66 ± 0.154
		22	184 ± 62.0	1080 ± 41.8	91.6 ± 14.5	NA	36.0 ± 1.39	3.05 ± 0.483
	Male	1	119 ± 15.1	840 ± 80.1	51.7 ± 9.21	77.7 ± 17.3	28.0 ± 2.67	1.72 ± 0.307
		22	177 ± 48.5	1050 ± 145	89.8 ± 18.5	NA	35.1 ± 4.82	2.99 ± 0.616

Data represents 5 NHP per gender per dose

*NA*  not applicable

The major pharmacodynamic effect was a reduction in peripheral B lymphocyte numbers observed within 24 h of APX005M administration in most animals dosed at 3 and 30 mg APX005M/kg (data not shown). This effect was observed over the course of the 28-day observation period following the final administration of APX005M. Since APX005M does not mediate ADCC and CDC on CD40 + cells but can activate B cells, it is possible that APX005M activated circulating B cells leading to extravasation of B cells to peripheral lymphoid tissues such as lymph nodes as has been hypothesized for other CD40 agonists [20]. There were no findings suggestive of adverse effects on organ system function including central nervous, respiratory, and cardiovascular systems, indicating that APX005M was well-tolerated in monkeys. While increases in several cytokines were observed in human PBMC cultures stimulated with APX005M, including TNF-α, IL-6, IL-12 and IFN-γ (Supplemental Fig. 2), there were no drug-associated changes in IL-1β, IL-2, IL-4, IL-5, IL 6, IL-12/23 (p40), IL-13, IL-17, G-CSF, GM-CSF, IFN-γ, and TNF-α in cyno monkey PBMC culture. Since the binding of APX005M to cyno CD40 is similar to human, these differences may be attributed to differences in Fc receptor expression between the species.

## Discussion

The treatment of cancer by manipulation of the immune system is a therapeutic modality with potentially curative effect. Antibodies targeting ICIs have shown significant and long-lasting anti-tumor activity in some patients, but many fail to respond to treatment or become refractory suggesting that simply blocking T-cell checkpoints is not sufficient to restore anti-tumor immunity in every case. One promising alternative to ICIs is the use of CD40 agonist antibodies to prime APCs and invigorate anti-tumor T cells that have been inhibited by the suppressive TME. In addition, CD40 agonism can potentially prime immune responses to novel T-cell epitopes further facilitating cancer eradication.

Agonistic antibodies tend to have different functionality and potency depending upon the affinity, epitope and FcR binding profile emphasizing the importance of careful selection of the appropriate binding epitope, affinity, and Fc engineering to achieve the desired profile of agonist antibodies for cancer immunotherapy. We demonstrate that APX005M is a potent CD40 agonist antibody that binds the CD40L binding site and depends on Fc receptor-mediated crosslinking for bioactivity. These properties make APX005M unique among the CD40 agonists currently in clinical development. APX005M potently activates APCs leading to expression of costimulatory molecules and inflammatory cytokine secretion followed by robust T-cell proliferation in response to antigens, including tumor-specific antigen. APX005M was the only agonist tested in our studies that induced potent secretion of IL-12 p70, which is essential for activation of naïve T cells and NK cells, indicating that APX005M may play an important role in initiating anti-tumor immune responses by multiple immune cell types. We hypothesize that the unique features of APX005M are necessary to enable in vivo priming of effective anti-tumor immune responses with less systemic toxicities.

Conflicting data have been published regarding the role of epitope specificity in conferring agonistic activity to CD40 antibodies. For example, it was recently suggested that CD40 agonist antibodies that bind membrane-distal epitopes of CD40 without blocking ligand binding are more potent agonists [22]. One hypothesis was that membrane-distal epitopes allow better access to the Fc portion of the antibody for more efficient cross-linking. Here, we show that APX005M, which binds to the CD40L binding site and effectively competes with CD40L, elicits more potent agonistic activity than antibodies that do not block ligand binding, suggesting Fc-crosslinking can still occur when the epitope on CD40 is proximal to the cell membrane. To the best of our knowledge, aside from APX005M, all other CD40 agonist antibodies in clinical development bind epitopes that do not compete with CD40L for binding [20, 38–40]. Our data argue that the specific binding epitope of APX005M may have biological and therapeutic significance when coupled with a requirement for FcR-dependent crosslinking. APX005M likely activates CD40 in a manner similar to endogenous CD40L because both bind overlapping sites on CD40 and likely have similar modes of action.

In addition to the unique epitope of APX005M, the Fc region of APX005M plays a critical role in mediating its functions, as F(ab’)2 fragments of APX005M lack biological activity. This contrasts with the Fc-independent activity seen following stimulation with CP-870,893 [22, 41] suggesting that the combination of binding epitope and interaction with FcRs is essential for optimal agonistic activity of CD40 agonist antibodies. Fitting with this hypothesis, the two commonly used anti-mouse CD40 agonist antibody clones, FGK45 and IC10, bind the CD40L binding site of CD40 and depend on FcR crosslinking and both show potent anti-tumor efficacy in preclinical mouse models [24]. These antibodies therefore represent the best mouse-specific surrogates for APX005M.

The requirement for Fc-crosslinking varies among CD40 agonist antibodies currently in clinical development. ADC-1013, SGN-40, and ChiLob 7/4 all require crosslinking for agonist activity whereas CP-870,893 agonist activity is independent of crosslinking [39, 41–43]. In mouse models, enhanced engagement of CD40 agonist antibodies by FcγRIIb results in optimal CD40 agonist activity [14, 23, 26]. As such, APX005M was optimized for engagement to FcγRIIb. Our data are consistent with studies that showing CD40 agonist activity is significantly enhanced by binding to FcγRIIb, which mediates crosslinking of the antibody leading to efficient clustering of CD40 receptors facilitating downstream signaling [23, 26]. In addition to enhancing the potency of APX005M, the S267E mutation reduces its binding to FcγRIIIa resulting in abrogation of ADCC activity. This feature of APX005M further enhances its therapeutic value by preventing the depletion of CD40-expressing APCs, which are key mediators of the anti-tumor response. The requirement for FcγR binding may translate into a better safety profile for APX005M in the clinic.

Because cells expressing FcγRIIb are most prevalent in tumor tissues and tumor draining lymph nodes (TDLNs), we hypothesize that APX005M may not be as active in the circulation due to limited FcR crosslinking. This should reduce on-target toxicities observed with other CD40 agonists, such as cytokine release syndrome. In contrast, TDLNs and tumor environment provides more cell–cell interaction promoting effective CD40 crosslinking through FcγRIIb [44] and allowing for maximum potency of APX005M in the tumor itself.

To better understand the pharmacology of APX005M and inform dosing regimen for first-in-human studies, we performed a correlation between receptor occupancy and activity. We found that maximal APX005M activity could be achieved at only 10% receptor occupancy, suggesting efficacy may be seen in humans at relatively low doses. In addition, we found that short-term exposure to APX005M resulted in long-term APC activation, suggesting that APX005M can be administered less frequently. Our initial results from a phase 1 dose-escalation study with APX005M (Study NCT02482168) revealed potent and dose-dependent on-target bioactivity demonstrated by the induction of peripheral immune activation based on cell phenotyping and serum cytokines at relatively low doses and with every 3 week dosing [28]. These data further validate the concept that APX005M stimulates immune activation in patients with cancer with a well tolerated safety profile

### Supplementary Information

Below is the link to the electronic supplementary material.

Supplementary file1 (PPTX 269 KB)

## Abbreviations

APC: Antigen presenting cell
ICA: Immune checkpoint activator
ICI: Immune checkpoint inhibitor
IRAE: Immune-related adverse events
PBMC: Peripheral blood mononuclear cells
