# Supplementary material for: APX005M, a CD40 agonist antibody with unique epitope specificity and Fc receptor binding profile for optimal therapeutic application
Source: Cancer Immunol Immunother. 2021 Jan 3;70(7):1853–65. doi: 10.1007/s00262-020-02814-2 (PMC8195934; doi:10.1007/s00262-020-02814-2)
Supplement: Supplementary file 1 — Supplementary file1 (PPTX 269 KB) [file 262_2020_2814_MOESM1_ESM.pptx]

## Slide 1
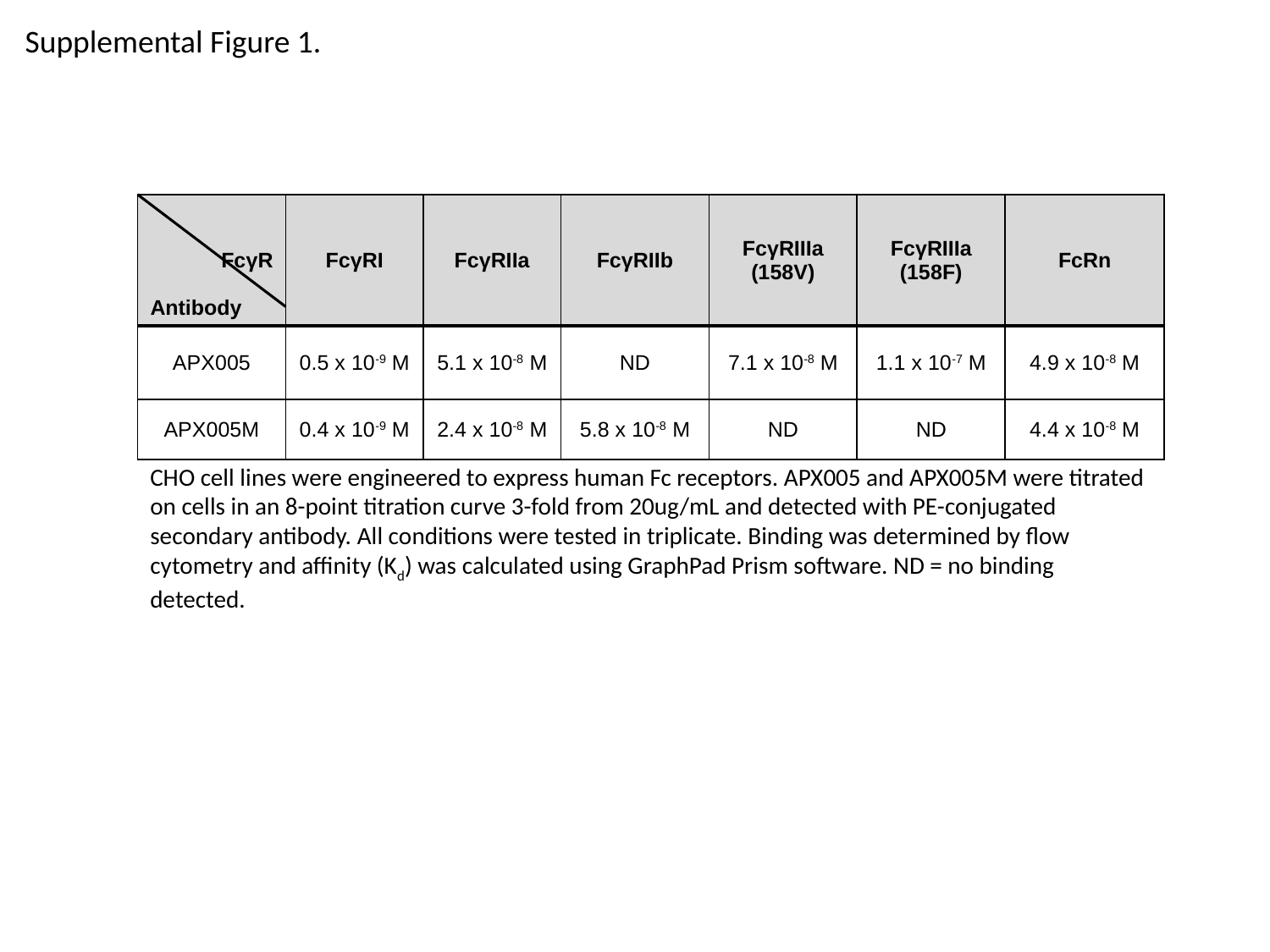

Supplemental Figure 1.
| FcγR Antibody | FcγRI | FcγRIIa | FcγRIIb | FcγRIIIa (158V) | FcγRIIIa (158F) | FcRn |
| --- | --- | --- | --- | --- | --- | --- |
| APX005 | 0.5 x 10^-9^ M | 5.1 x 10^-8^ M | ND | 7.1 x 10^-8^ M | 1.1 x 10^-7^ M | 4.9 x 10^-8^ M |
| APX005M | 0.4 x 10^-9^ M | 2.4 x 10^-8^ M | 5.8 x 10^-8^ M | ND | ND | 4.4 x 10^-8^ M |
CHO cell lines were engineered to express human Fc receptors. APX005 and APX005M were titrated on cells in an 8-point titration curve 3-fold from 20ug/mL and detected with PE-conjugated secondary antibody. All conditions were tested in triplicate. Binding was determined by flow cytometry and affinity (Kd) was calculated using GraphPad Prism software. ND = no binding detected.

## Slide 2
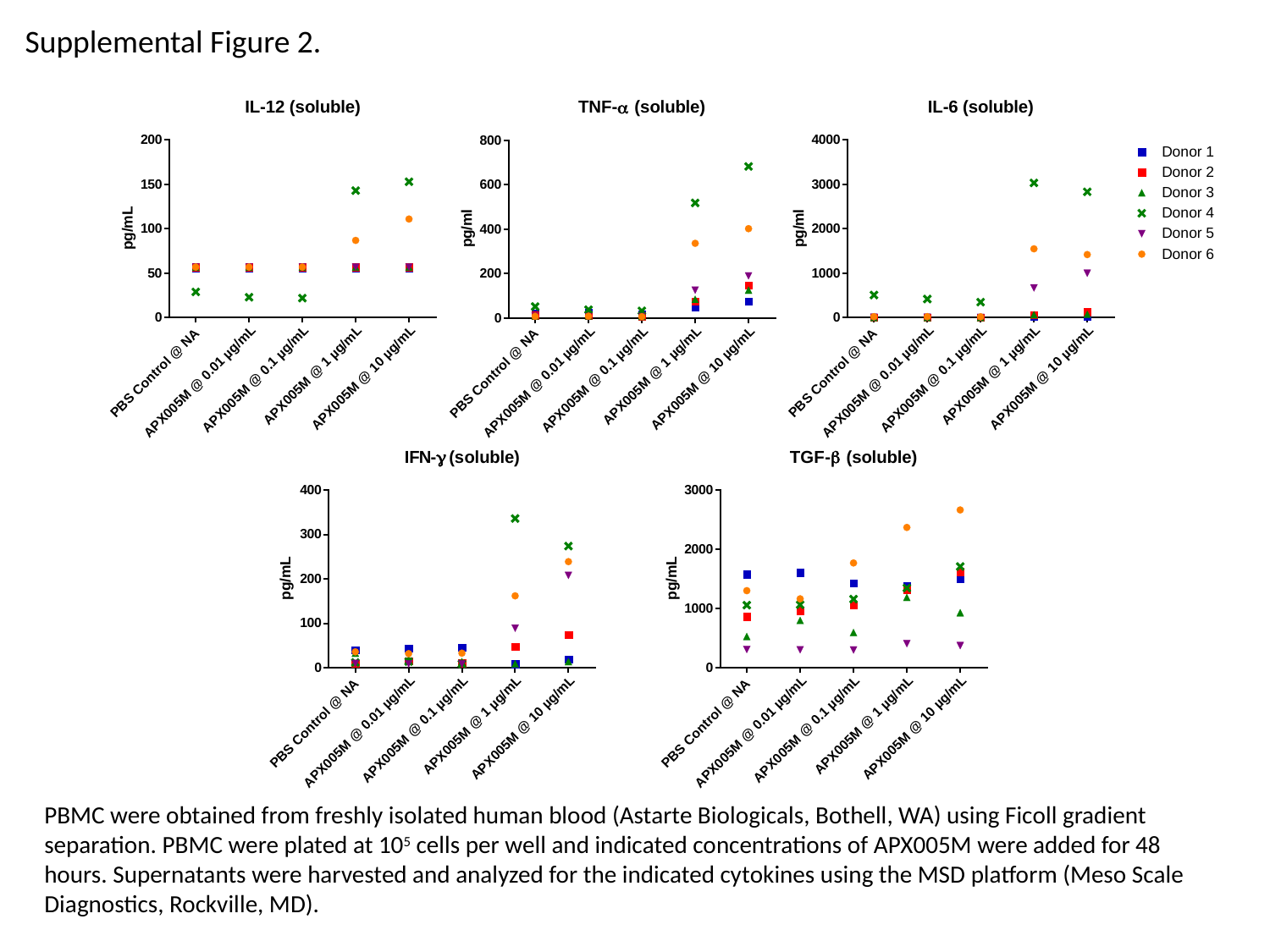

Supplemental Figure 2.
PBMC were obtained from freshly isolated human blood (Astarte Biologicals, Bothell, WA) using Ficoll gradient separation. PBMC were plated at 105 cells per well and indicated concentrations of APX005M were added for 48 hours. Supernatants were harvested and analyzed for the indicated cytokines using the MSD platform (Meso Scale Diagnostics, Rockville, MD).
